# Nocturia independently predicts left ventricular hypertrophy and left atrial enlargement among patients with cardiac symptoms

**DOI:** 10.1038/s41598-022-19190-9

**Published:** 2022-09-01

**Authors:** Kuo-Wei Kao, Weiming Cheng, Ching-Ju Wu, Yu-Hua Fan

**Affiliations:** 1 Division of Urology, Department of Surgery, Taipei City Hospital, Renai Branch, Taipei, Taiwan; 2 Division of Urology, Department of Surgery, Taipei City Hospital, Zhongxiao Branch, Taipei, Taiwan; 3 Institute of Biopharmaceutical Science, College of Life Science, National Yang Ming Chiao Tung University, Taipei, Taiwan; 4 Department of Urology, College of Medicine, National Yang Ming Chiao Tung University, Taipei, Taiwan; 5 Program in Molecular Medicine, College of Life Sciences, National Yang Ming Chiao Tung University, Taipei, Taiwan; 6 Division of Cardiology, Department of Medicine, Taipei City Hospital, Zhongxiao Branch, Taipei, Taiwan; 7 Department of Urology, Taipei Veterans General Hospital, Taipei, Taiwan

**Keywords:** Cardiology, Medical research, Signs and symptoms, Urology

## Abstract

Nocturia can be caused by urological disorders and systemic diseases, including heart diseases. We aimed to investigate the relationship between nocturia and structural abnormalities on echocardiography. Adult patients who underwent echocardiography for cardiac symptoms or heart murmur or had a history of structural heart disease were included. The voiding times during sleep hours were collected prospectively. Univariate and multivariate analyses were performed to evaluate the predictive value of bothersome nocturia (nocturia ≥ 2) on echocardiographic abnormalities. Of 299 patients, 182 (60.9%) reported bothersome nocturia. In patients aged ≥ 65 years, hypertension and left atrial enlargement (LAE) were associated with higher occurrences of bothersome nocturia. On multivariate analysis, bothersome nocturia was a predictive factor of LAE (odds ratio [OR] 2.453, 95% confidence interval [CI] 1.363–4.416, *p* = 0.003). Moreover, bothersome nocturia could predict both LAE and left ventricular hypertrophy (LVH) (OR 2.285, 95% CI 1.151–4.536, *p* = 0.018; OR 2.056, 95% CI 1.025–4.124, *p* = 0.043) in the elderly. Older age, hypertension, and LAE were risk factors for bothersome nocturia. Moreover, bothersome nocturia was predictive of LAE and LVH in the elderly. Patients with bothersome nocturia without other significant lower urinary tract symptoms should be referred to cardiologists.

## Introduction

Nocturia is the most common and troublesome lower urinary tract symptom (LUTS)^1^. Nocturia prevalence ranges from 11 to 35.2% in young men and 20.4% to 43.9% in young women. Moreover, the prevalence is between 68.9 and 93% in the elderly population^2^. Nocturia is associated with poor quality of life and increased morbidity^3^. Furthermore, nocturia is strongly associated with depression^4^ and leads to a higher risk of incident falls in the elderly^5^. In addition to urological disorders, nocturia is also caused by systemic diseases^6^. Previous studies have reported that heart failure and uncontrolled hypertension were responsible for clinically significant nocturia^7,8^. Other cardiac diseases may also contribute to nocturia. One study revealed that electrocardiographic evidence of left ventricular hypertrophy (LVH) and left atrial enlargement (LAE) were significantly associated with nocturia^9^. However, to the best of our knowledge, no studies have so far thoroughly evaluated the association between structural cardiac abnormalities and nocturia. We, thus, investigated the relationship between nocturia and structural abnormalities on cardiac sonography.

## Materials and methods

The study was approved by the Institutional Review Board of Taipei City Hospital (approval number: TCHIRB-11002002) and performed in accordance with the guidelines of the Declaration of Helsinki (as revised in 2013). Written informed consent was obtained from all patients.

We prospectively included adult patients from the cardiology outpatient department who underwent cardiac sonography between June 1, 2021, and July 31, 2021. Cardiac sonography was undertaken for clinical symptoms like chest pain or tightness, physical examination with heart murmur, or follow-up of structural heart disease. Patients with urinary tract infection, neurogenic bladder, or a prior history of lower urinary tract or pelvic surgery were excluded. Before the echocardiographic examination, nocturia was assessed using Question 7 of the International Prostate Symptom Score (IPSS) questionnaire “How many times did you typically get up at night to urinate?”. The patients’ basic data included age, body mass index (BMI), and presence of comorbidities, such as diabetes mellitus, hypertension, heart failure and cardiomyopathy. Elderly patients were defined as those aged ≥ 65 years. BMI ≥ 25 kg/m^2^ was defined as obesity in the Asian population^10^.

The 2018 International Continence Society (ICS) defines nocturia as waking for passing urine during the main sleep hours^11^. Previous literature showed that the majority of people report being troubled when the number of nocturia episodes is ≥ 2 and that over two voids per night is associated with impaired health-related quality of life^12^. Therefore, we classified the participants into two groups based on the number of nocturia episodes: bothersome nocturia (nocturia ≥ 2) vs non-bothersome nocturia (nocturia ≤ 1).

Cardiac sonography was performed by a single technician and interpreted by a cardiologist. A cardiologist has validated this cardiac sonography report. All three cardiac staff were blinded to the patients’ nocturia conditions. The performance and measurements of echocardiographic parameters were based on the 2015 recommendations of chamber quantification of the American Society of Echocardiography (ASE) and the European Association of Cardiovascular Imaging (EACVI)^13^. Echocardiological parameters, including the size of the left atrium (LA), left ventricle (LV), aorta, aortic valve (AV), mitral valve (MV), tricuspid valve (TV), and pulmonary valve (PV), were measured. The aortic valve sinus diameter, interventricular septal thickness at end-diastole (IVSd), left ventricular posterior wall thickness at end-diastole (LVPWd), left ventricular internal diameter in diastole (LVIDd), and left ventricular internal diameter in systole (LVIDs) were recorded. Left ventricular systolic and diastolic function and valve regurgitation were also measured using Doppler echocardiography.

Left ventricular ejaction fraction (LVEF) was calculated using the Simpson’s biplane method. In males, the normal LVEF range was 52–72%, mildly abnormal was 41–51%, moderately abnormal was 30–40%, and severely abnormal was less than 30%. In females, the normal range was 54–74%, mildly abnormal was 41–53%, moderately abnormal was 30–40%, and severely abnormal was less than 30%^13^. LV diastolic dysfunction (LVDD) was measured by mitral valve E velocity divided by A-wave velocity (E/A ratio) and mitral valve E velocity divided by mitral annular e′velocity (average E/e’ ratio), and the normal range was E/A ≥ 0.8 and E/e^’^ ratio < 10, grade I was E/A ≤ 0.8 and E/e^’^ ratio < 10, grade II was E/A > 0.8 to < 2 and E/e^’^ ratio 10–14, and grade III was E/A > 2 and E/e^’^ ratio > 14^14^. Left atrial enlargement (LAE) was defined as a left atrial dimension of > 40 mm^15^. Aortic root dilation was defined as an aortic valve sinus diameter > 38 mm in men and > 36 mm in women^16^. Left ventricular mass (LVM) was calculated using the following formula: LVM = 0.8 × [1.04 × {(LVIDd + LVPWd + IVSd)^3^- (LVIDd)^3^}] + 0.6 g^17^. The left ventricular mass index (LVMI) was calculated by normalizing the LVM by the body surface area based on Devereux’s formula^18^. Left ventricular hypertrophy (LVH) was defined as an increased left ventricular mass index (LVMI) (≥ 125 g/m^2^ in men and ≥ 110 g/m^2^ in women)^19^. Concentric LV remodeling was defined as LVMI ≤ 115 g/m^2^ in men or ≤ 95 g/m^2^ in women and LV relative wall thickness (RWT) > 0.42^20^. Moderate-to-severe AV regurgitation (AR) was considered to have intermediate-to-large color flow in AR jet width, increment vena contracta width (> 3 mm), and a decrease in pressure half-time (< 500 ms). Moderate-to-severe TV regurgitation (TR), MV regurgitation (MR), and PV regurgitation (PR) were defined as intermediate-to-large color flows in the TR, MR, and PR jet widths and dense continuous wave signals of the TR, MR, and PR jets, respectively^21^.

Statistical analyses were performed using IBM SPSS Statistics for Macintosh, ver. 24 (IBM Corp., Armonk, NY, USA). Pearson’s chi-square test was used to analyze factors associated with bothersome nocturia. Univariate and multivariate analyses were performed with logistic regression to evaluate the predictive value of bothersome nocturia on echocardiographic abnormalities after adjusting for sex, age, obesity, hypertension, diabetes mellitus, diuretic use and heart disease. Statistical significance was set at *p* < 0.05.

## Results

A total of 299 patients aged between 20 and 95 years were included in the study, of whom 134 (44.8%) were men and 165 (55.2%) were women. Elderly patients accounted for 70.6%, while patients with obesity accounted for 50.8%. Hypertension was found in 226 (75.6%) patients, and 4 (1.3%) patients had a history of heart diseases, including three with heart failure and one with cardiomyopathy. Bothersome nocturia was reported in 182 patients (60.9%). Regarding echocardiographic abnormalities, 27 (9%) had abnormal LVEF, 10 (3.3%) had grade II-III LV diastolic dysfunction, 109 (36.5%) had LAE, and 85 (28.4%) patients had LVH (Table 1).

**Table 1:** Patient demographic characteristics and echocardiographic findings

	Patients (N = 299)	
	Number	Percentage (%)
**Age**		
< 65 years	88	29.4
≥ 65 years	211	70.6
**Sex**		
Male	134	44.8
Female	165	55.2
**BMI**		
< 25	147	49.2
≥ 25	152	50.8
**Bothersome nocturia**		
Yes	182	60.9
No	117	39.1
**Hypertension history**		
Yes	226	75.6
No	73	24.4
**Heart disease history**		
Yes	4	1.3
No	299	98.7
**Diuretic usage**		
Yes	17	5.7
No	282	94.3
**Diabetes mellitus**		
Yes	93	31.1
No	206	68.9
**Aortic root dilation**		
Yes	24	8
No	275	92
**Left atrial enlargement**		
Yes	109	36.5
No	190	63.5
**Left ventricle hypertrophy**		
Yes	85	28.4
No	214	71.6
**Concentric remodeling**		
Yes	122	40.8
No	177	59.2
**Moderate-to-severe AR**		
Yes	62	20.7
No	237	79.3
**Moderate-to-severe TR**		
Yes	107	35.8
No	192	64.2
**Moderate-to-severe MR**		
Yes	79	26.4
No	220	73.6
**Moderate-to-severe PR**		
Yes	59	19.7
No	240	80.3
**Left ventricular ejection fraction**		
Abnormal	27	9
Normal	272	91
**Left ventricular diastolic dysfunction**		
Grade II to III	10	3.3
Normal or grade I	289	96.7
**Stenotic valvular disorder**		
Yes	1	0.3
No	289	99.7

*AR* aortic valve regurgitation; *TR* tricuspid valve regurgitation; *MR* mitral valve regurgitation;

*PR* pulmonary valve regurgitation.

Among all patients, those older than 65 years of age were found to have higher occurrences of bothersome nocturia (69.2% vs. 40.9%, *p* < 0.001). Patients with hypertension (64.2% vs. 50.7%, *p* = 0.040) and LAE (73.4% vs. 53.7%, *p* = 0.001) also had higher occurrences of bothersome nocturia. Additionally, there was a tendency for patients with LVH to have higher occurrences of bothersome nocturia with a borderline significance (69.4% vs. 57.5%, *p* = 0.056) (Table 2).

**Table 2:** Difference in the prevalence of bothersome nocturia among all patients and elderly patients (≥ 65 years).

		All (N = 299)			Elderly patients (N = 211)		
		Non-bothersome nocturia, N(%)	Bothersome nocturia, N(%)	*p*-value	Non-bothersome Nocturia, N (%)	Bothersome Nocturia, N (%)	*p*-value
**Age ≥ 65**							
	No	52 (59.1%)	36 (40.9%)	** < 0.001**			
	Yes	65 (30.8%)	146 (69.2%)				
**Age ≥ 80**							
	No				52 (33.5%)	103 (66.5%)	0.151
	Yes				13 (23.2%)	43 (76.8%)	
**Sex**							
	Male	52 (38.8%)	82 (61.2%)	0.917	24 (27.0%)	65 (73.0%)	0.302
	Female	65 (39.4%)	100 (60.6%)		41 (33.6%)	81 (66.4%)	
**Obesity**							
	No	53 (36.1%)	94 (63.9%)	0.284	26 (24.3%)	81 (75.7%)	**0.038**
	Yes	64 (42.1%)	88 (57.9%)		39 (41.9%)	54 (58.1%))	
**Hypertension history**							
	No	36 (49.3%)	37 (50.7%)	**0.040**	14 (32.6%)	29 (67.4%)	0.780
	Yes	81 (35.8%)	145 (64.2%)		51 (30.4%)	117 (69.6%)	
**Diuretic use**							
	No	110 (39.0%)	172 (61.0%)	0.859	62 (31.2%)	137 (68.8%)	0.759
	Yes	7 (41.2%)	10 (58.8%)		3 (25.0%)	9 (75.0%)	
**Diabetes mellitus history**							
	No	87 (42.2%)	119 (57.8%)	0.102	47 (32.6%)	97 (67.4%)	0.398
	Yes	30 (25.6%)	63 (34.6%)		18 (26.9%)	49 (73.1%)	
**Aortic root dilatation**							
	No	104 (37.8%)	171 (62.2%)	0.116	56 (29.0%)	137 (71.0%)	0.106
	Yes	13 (54.2%)	11 (45.8%)		9 (50.0%)	9 (50.0%)	
**Left atrial enlargement**							
	No	88 (46.3%)	102 (53.7%)	**0.001**	46 (36.8%)	79 (63.2%)	**0.024**
	Yes	29 (26.6%)	80 (73.4%)		19 (22.1%)	67 (77.9%)	
**Left ventricle hypertrophy**							
	No	91 (42.5%)	123 (57.5%)	0.056	49 (35.0%)	91 (65.0%)	0.064
	Yes	26 (30.6%)	59 (69.4%)		16 (22.5%)	55 (77.5%)	
**Concentric remodeling**							
	No	74 (41.8%)	103 (58.2%)	0.253	35 (30.7%)	79 (69.3%)	0.972
	Yes	43 (35.2%)	79 (64.8%)		30 (30.9%)	67 (69.1%)	
**Moderate-to-severe AR**							
	No	97 (40.9%)	140 (59.1%)	0.213	52 (32.1%)	110 (67.9%)	0.459
	Yes	20 (32.3%)	42 (67.7%)		13 (26.5%)	36 (73.5%)	
**Moderate-to-severe TR**							
	No	74 (38.5%)	118 (61.5%)	0.780	36 (28.1%)	92 (71.9%)	0.295
	Yes	43 (40.2%)	64 (59.8%)		29 (34.9%)	54 (65.1%)	
**Moderate-to-severe MR**							
	No	89 (40.5%)	131 (59.5%)	0.434	46 (30.7%)	104 (69.3%)	0.945
	Yes	28 (35.4%)	51 (64.6%)		19 (31.1%)	42 (68.9%)	
**Moderate-to-severe PR**							
	No	91 (37.9%)	149 (62.1%)	0.386	49 (29.7%)	116 (70.3%)	0.509
	Yes	26 (44.1%)	33 (55.9%)		16 (34.8%)	30 (65.2%)	
**LV ejection fraction**							
	Normal	108 (39.7%)	164 (60.3%)	0.518	63 (32.1%)	133 (67.9%)	0.156
	Abnormal	9 (33.3%)	18 (66.7%)		2 (13.3%)	13 (86.7%)	
**LV diastolic dysfunction**							
	Normal or grade I	114 (39.4%)	175 (60.6%)	0.745	62 (30.8%)	139 (69.2%)	1.000
	Grade II to III	3 (30.0%)	7 (70.0%)		3 (30.0%)	7 (70.0%)	

*AR* aortic valve regurgitation; *TR* tricuspid valve regurgitation; *MR* mitral valve regurgitation; *PR* pulmonary valve regurgitation; *LV* Left ventricular.

In elderly patients, the prevalence of bothersome nocturia was not associated with increasing age and the presence of hypertension. Similarly, patients with LAE had higher occurrences of bothersome nocturia (77.9% vs. 63.2%, *p* = 0.024), and those with LVH tended to have higher occurrences of bothersome nocturia with a borderline significance (77.5% vs. 65.0%, *p* = 0.064). Furthermore, the prevalence of bothersome nocturia was lower in obese patients compared with that in non-obese patients (58.1% vs. 75.7%, *p* = 0.038) (Table 2).

On multivariate analysis, in addition to age, sex, obesity, hypertension, diabetes mellitus, use of diuretics and heart disease, we also adjusted for LVH in the evaluation of the predictive value of bothersome nocturia on LAE because LVH and LAE are related to each other with respect to diastolic dysfunction. Bothersome nocturia was a predictive factor of LAE (odds ratio [OR], 2.453; 95% confidence interval [CI], 1.363–4.416; *p* = 0.003). Moreover, in the subgroup of patients older than 65 years, bothersome nocturia was predictive of LAE (OR, 2.285; 95% CI, 1.151–4.536; *p* = 0.018) and LVH (OR, 2.056; 95% CI, 1.025–4.124, *p* = 0.043) (Table 3).

**Table 3:** Multivariate analysis to investigate the predictive value of bothersome nocturia on various echocardiographic abnormalities in all patients and in the elderly (≥ 65 years) patient groups.

Structural heart diseases	All patients				Elderly patients			
	Odds ratio of bothersome nocturia	95% CI		*p*-value	Odds ratio of bothersome nocturia	95% CI		*p*-value
Left atrial enlargement	2.453^a^	1.363	4.416	**0.003**	2.285 ^a^	1.151	4.536	**0.018**
Left ventricle hypertrophy	1.609	0.882	2.936	0.121	2.056	1.025	4.124	**0.043**
Moderate-to-severe AR	1.309	0.696	2.465	0.404	1.190	0.56	2.527	0.651
Moderate-to-severe TR	0.846	0.495	1.446	0.541	0.597	0.313	1.142	0.119
Moderate-to-severe MR	1.211	0.677	2.165	0.519	0.943	0.478	1.86	0.866
Moderate- to-severe PR	0.68	0.359	1.287	0.236	0.693	0.333	1.441	0.326

*AR* aortic valve regurgitation; *TR* tricuspid valve regurgitation; *MR* mitral valve regurgitation; *PR* pulmonary valve regurgitation.

^a^Besides adjustment with patients’ age, sex, obesity, hypertension, diabetes mellitus, diuretic use, and, heart disease in other echocardiographic abnormalities, left ventricle hypertrophy was also added to adjust for left atrial enlargement.

Significant Values are in bold.

## Discussion

In the present study, we found that old age, hypertension, and echocardiographic evidence of LAE were significantly associated with a higher prevalence of bothersome nocturia. Moreover, bothersome nocturia was predictive of LAE and LVH, especially in the elderly. To the best of our knowledge, this is the first study to correlate nocturia with echocardiographic evidence of structural heart diseases. Urologists should consider referring patients with bothersome nocturia to cardiologists to evaluate the presence of LAE or LVH.

Nocturia is closely associated with cardiac complications. Nocturia may be a risk factor for coronary heart disease in young men and increases the possibility of death in older men^22^. Left atrial enlargement is the most significant cardiac abnormality associated with nocturia. This connection has been documented previously and is believed to result from the elevation of atrial natriuretic peptide (ANP) levels in patients with LAE^23^. A previous study showed that increased ANP due to subclinical heart failure can cause nocturnal urinary symptoms^24^. ANP increases the glomerular filtration rate (GFR), renal plasma flow, and sodium excretion, which causes diuresis and natriuresis^25^. Another possible mechanism is that LAE leads to cardiac structural changes and causes sodium and water retention in the extracellular space^26^. When sleeping in a supine position, the redistribution of fluid from edematous legs may cause nocturnal polyuria^27^.

Bothersome nocturia was also predictive of LVH in patients older than 65 years. Left ventricular hypertrophy increases the risk of both systolic and diastolic heart failure^27^. Congestive heart failure may decrease renal plasma flow, increase the filtration fraction during ambulation, and cause sodium retention. Nighttime sleep in the supine position improves renal hemodynamics and sodium excretion, leading to nocturia^28^.

We found that bothersome nocturia was more prevalent among older adults. This is consistent with the results of previous studies^29,30^. The incidence and severity of nocturia increase with age in both men and women^31^. Urological problems and systemic diseases affect nocturia in older patients equally, including bladder storage problems, polyuria, nocturnal polyuria, and sleep-related issues^32^. Nocturia in the elderly leads to impaired quality of life, for both the patients and their cohabitants, and increases the incidence of falls, resulting in higher morbidity^30^. Interventions such as behavioral, pharmacological, or surgical management should be considered to improve nocturia severity in the elderly^33^.

Our study reported that the prevalence of bothersome nocturia was lower in obese patients compared with that in non-obese patients in the elderly patient group, implying a protective role of obesity in the development of bothersome nocturia. In contrast, previous studies demonstrated a positive association between obesity and nocturia. A study that included approximately 3,600 patients in Finland concluded that obesity is strongly associated with increased nocturia^34^. Another study that analyzed 14135 patients from the National Health and Nutrition Examination Survey in the United States showed that obesity is significantly associated with nocturia^35^. Our results, which differ from previous studies, may be due to the differences in race, age, and study population. We focused on elderly patients with cardiac symptoms and potential structural heart disease, which may have a stronger negative effect than obesity on nocturia and modify the effect of obesity on nocturia. Moreover, the definitions of obesity based on BMI also differ between Asians and Caucasians^36^. These disparities could have contributed to the different results.

Our study showed a significant relationship between hypertension and bothersome nocturia. Uncontrolled hypertension and elevation of blood pressure are reportedly related to nocturia^9,37^. Hypertension affects renal glomerular filtration and tubular transport. In addition, hypertension can increase renal plasma flow and decrease sodium reabsorption, leading to nocturia^38,39^. A previous study indicated a significant relationship between diabetes mellitus and nocturia^40^. Hyperglycemia and osmotic diuresis occur in patients with diabetes mellitus, predisposing them to nocturia^41^. Proper control of serum glucose levels in diabetic patients helps improve nocturia^41^. However, in this study, a history of diabetes mellitus was not significantly associated with bothersome nocturia. One possible explanation is that all patients included in our study received appropriate treatment for blood sugar.

Our study had several limitations. First, we included patients from the cardiology department rather than the general population. The association between bothersome nocturia and echocardiographic abnormalities, especially in those without obvious cardiac symptoms, may be underestimated. Second, although this was a prospective study, we did not analyze the effects of antihypertensive drugs other than diuretics on nocturia. Third, we did not perform prostate ultrasonography to evaluate the size of the prostate gland in male participants, which may also have an impact on the severity of nocturia. Fourth, we did not follow-up with the patients in a cross-sectional study to evaluate the association between nocturia and echocardiographic abnormalities. Fifth, we evaluated LA size using conventional M-mode LA dimension rather than LA volume. Measurement of LA size using conventional M-mode LA dimension is simple and convenient but not reliably accurate, given that the LA is not a symmetrically shaped three-dimensional structure. It is unknown whether treatment of structural heart diseases, such as LAE or LVH, could also improve the severity of nocturia. Nevertheless, the results of this study have important implications. Our results suggest that urologists should refer patients with bothersome nocturia without other significant LUTS to cardiologists to evaluate the presence of structural heart disease, which may be potentially critical to patients.

## Conclusion

Older age, hypertension, and echocardiographic evidence of LAE are associated with a higher incidence of bothersome nocturia. Furthermore, bothersome nocturia could predict structural heart diseases, including LAE and LVH, in the elderly. Referral to cardiologists should be considered for patients with bothersome nocturia.

## Data Availability

Data utilized in our study are available from the Taipei City Hospital. Based on the “Personal Information Protection Act” in Taiwan, data cannot be made publicly available due to legal restrictions imposed by the government of Taiwan. Requests for data can be sent as a formal proposal to the Institutional Review Board of Taipei City Hospital.
